# Dual Roles of GSNOR1 in Cell Death and Immunity in Tetraploid *Nicotiana tabacum*

**DOI:** 10.3389/fpls.2021.596234

**Published:** 2021-02-10

**Authors:** Zhen-Chao Li, Qian-Wei Ren, Yan Guo, Jie Ran, Xiao-Tian Ren, Ni-Ni Wu, Hui-Yang Xu, Xia Liu, Jian-Zhong Liu

**Affiliations:** ^1^College of Chemistry and Life Sciences, Zhejiang Normal University, Jinhua, China; ^2^Zhejiang Provincial Key Laboratory of Biotechnology on Specialty Economic Plants, Zhejiang Normal University, Jinhua, China

**Keywords:** cell death, disease resistance, nitric oxide, hypersensitive responses, reactive oxygen species, S-nitrosoglutathione reductase

## Abstract

S-nitrosoglutathione reductase 1 (GSNOR1) is the key enzyme that regulates cellular homeostasis of *S-*nitrosylation. Although extensively studied in *Arabidopsis*, the roles of GSNOR1 in tetraploid *Nicotiana* species have not been investigated previously. To study the function of *Nt*GSNOR1, we knocked out two *NtGSNOR1* genes simultaneously in *Nicotiana tabacum* using clustered regularly interspaced short palindromic repeats (CRISPR)/caspase 9 (Cas9) technology. To our surprise, spontaneous cell death occurred on the leaves of the CRISPR/Cas9 lines but not on those of the wild-type (WT) plants, suggesting that *Nt*GSNOR1 negatively regulates cell death. The natural cell death on the CRISPR/Cas9 lines could be a result from interactions between overaccumulated nitric oxide (NO) and hydrogen peroxide (H_2_O_2_). This spontaneous cell death phenotype was not affected by knocking out two *Enhanced disease susceptibility 1* genes *(NtEDS11a/1b)* and thus was independent of the salicylic acid (SA) pathway. Unexpectedly, we found that the *NtGSNOR1a/1b* knockout plants displayed a significantly (*p* < 0.001) enhanced resistance to paraquat-induced cell death compared to WT plants, suggesting that *Nt*GSNOR1 functions as a positive regulator of the paraquat-induced cell death. The increased resistance to the paraquat-induced cell death of the *NtGSNOR1a/1b* knockout plants was correlated with the reduced level of H_2_O_2_ accumulation. Interestingly, whereas the *N* gene-mediated resistance to *Tobacco mosaic virus* (TMV) was significantly enhanced (*p* < 0.001), the resistance to *Pseudomonas syringae* pv. *tomato* DC3000 was significantly reduced (*p* < 0.01) in the *NtGSNOR1a/1b* knockout lines. In summary, our results indicate that *Nt*GSNOR1 functions as both positive and negative regulator of cell death under different conditions and displays distinct effects on resistance against viral and bacterial pathogens.

## Introduction

Nitric oxide (NO) is a reactive free radical gas molecule with a plethora of functions in both animals and plants (Wendehenne et al., 2014). In plants, NO is involved in diverse biological processes such as stomatal closure (Desikan et al., 2002; Neill et al., 2008), cell death and disease resistance (Durner et al., 1998; Klessig et al., 2000; Wendehenne et al., 2004; Zeidler et al., 2004; Lin et al., 2012; Wang et al., 2013; Wang and Chu, 2020), abiotic stress (Lee et al., 2008; Xuan et al., 2010), flowering (He et al., 2004), growth/development (Yun et al., 2016), and many other processes (Lamattina et al., 2003; Wendehenne et al., 2014). Among all these processes, the most significant function of NO is to potentiate the induction of hypersensitive cell death by reactive oxygen species (ROS) (Durner et al., 1998; Delledonne et al., 2001).

*S*-nitrosylation, attachment of NO moiety to a target protein, is a newly emerged mechanism by which NO regulates the function of various target proteins and thus various biological processes (Hess and Stamler, 2012; Wendehenne et al., 2014). This reversible posttranslational modification is analogous to protein phosphorylation (Stamler et al., 1992; Hess et al., 2005). Hundreds of proteins have been identified as targets of *S*-nitrosylation whose functions are regulated by this posttranslational modification (Lindermayr et al., 2005; Forrester et al., 2009; Hess and Stamler, 2012; Yang et al., 2015). In plants, the *S*-nitrosylated cysteine residues of some target proteins have been identified, and the functions of this posttranslational modifications are revealed (Lindermayr et al., 2006, 2010; Romero-Puertas et al., 2007; Tada et al., 2008; Yun et al., 2011; Skelly et al., 2019; Gupta et al., 2020).

*S*-nitrosoglutathione (GSNO), *S*-nitrosylated form of glutathione (GSH), functions as a major mediator of protein *S*-nitrosylation by a process known as transnitrosylation (Hess et al., 2005). S-nitrosoglutathione reductase (GSNOR) is the key enzyme controlling GSNO levels by reducing GSNO to oxidized GSH and NH_3_ and thus indirectly controlling the levels of protein *S*-nitrosylation (Liu et al., 2001, 2004; Feechan et al., 2005). In mice, loss of GSNOR1 function results in substantial increases in whole-cell S-nitrosylation, tissue damage, and mortality following endotoxic or bacterial challenge (Liu et al., 2004). In *Arabidopsis*, the hypersensitive response (HR) cell death triggered by avirulent *Pseudomonas syringae* pv. tomato DC3000 (*Pst* DC3000) carrying the *AvrB* or *AvrRPS4* is accelerated in loss of function mutant, *gsnor1-3*, but is delayed in overexpressing mutant *gsnor1-1* (Yun et al., 2011), suggesting that the function of GSNOR1 is conserved between mice and *Arabidopsis* in negatively regulating HR cell death in response to biotic stress (Liu et al., 2004; Yun et al., 2011). The cell death in response to incompatible pathogen infections in *gsnor1-3* mutant is due to high SNO concentrations and is SA- and hydrogen peroxide (H_2_O_2_)-independent (Yun et al., 2011). Interestingly, it has also been revealed that GSNOR1 is a key positive regulator of cell death induced by herbicides (Chen et al., 2009), suggesting that depending on cellular conditions, GSNOR1 could regulate cell death either positively or negatively. NO has been demonstrated to have both proapoptotic and antiapoptotic roles, depending on a variety of factors, including the type of cells involved, redox state of the cell, and the flux and dose of NO (Wang et al., 2013; Wendehenne et al., 2014; Huang et al., 2019). In general, *S*-nitrosylation resulting from basal low-level NO production in cells has antiapoptotic effects, whereas *S*-nitrosylation resulting from higher-level stimulated NO production has either proapoptotic effects or serves as a negative feedback mechanism to downregulate apoptotic responses (Mannick, 2007).

NO exerts its effects on cell death mostly through modulating H_2_O_2_ production or detoxification. *S*-nitrosylation of an *Arabidopsis* cytosolic ascorbate peroxidase (cAPX), a key enzyme controlling H_2_O_2_ levels in plants, inhibits its enzyme activity, leading to Programmed cell death (PCD) (de Pinto et al., 2013). Loss of H_2_O_2_ detoxifying enzyme, catalase in rice, results in both NO and H_2_O_2_ overaccumulation and leaf cell death (Lin et al., 2012). Overexpression of rice *GSNOR1* alleviates the leaf cell death in *noe1 (nitric oxide excess 1*) mutant (Lin et al., 2012), suggesting a conserved role of *S*-nitrosylation in cell death in plants. *S*-nitrosylation not only can induce cell death but also can function as a negative feedback loop to limit or prevent excessive cell death. Instead of inhibiting the function of a cAPX1, *S*-nitrosylation of the *Arabidopsis* cytosolic APX1 at Cys^32^ enhances its enzymatic activity of scavenging H_2_O_2_, resulting in an increased resistance to oxidative stress (Yang et al., 2015). NADPH oxidase is the major source of H_2_O_2_ production under biotic stress. *Arabidopsis* AtRBOHD subunit of NADPH oxidase complex is *S*-nitrosylated at Cys^890^, and this modification abolishes its ability to synthesize ROS and thus avoids excessive cell death (Yun et al., 2011).

In addition to cell death, GSNOR1 plays a critical role in disease resistance. Loss of GSNOR1 function in *Arabidopsis* results in compromised basal resistance, R gene-mediated resistance and non-host resistance (NHR) (Feechan et al., 2005). Silencing *SlGSNOR1* in tomato also compromises basal resistance against *Pst* DC3000 (Hussain et al., 2019). On the contrary, *Arabidopsis* and tomato lines that overexpress *GSNOR1* lead to enhanced resistance to virulent pathogens (Feechan et al., 2005; Hussain et al., 2019). However, silencing *GSNOR1* by antisense approach in *Arabidopsis* enhances Systemic acquired resistance (SAR) and basal resistance (Rustérucci et al., 2007). Recently, it has been shown that the accelerated cell death observed in *gsnor1-3* in response to pathogen infection confers *RPP4*-mediated resistance to *Hyaloperonospora arabidopsidis* isolate Emwa1 in an SA-independent manner (Yun et al., 2011). These seemingly contradictory results suggest that the roles of GSNOR1 in plant disease resistance are more complicated than we thought. Besides cell death and disease resistance, GSNOR1 is also required for growth/development (Feechan et al., 2005; Lee et al., 2008; Chen et al., 2009; Albertos et al., 2015; Shi et al., 2015; Yun et al., 2016; Kawabe et al., 2018; Gong et al., 2019; Hussain et al., 2019), thermotolerance (Lee et al., 2008), abiotic stress responses (Zhang and Liao, 2019), hypoxia responses (Zhan et al., 2018), cytokinin signaling (Feng et al., 2013), auxin signaling (Terrile et al., 2012; Shi et al., 2015; Ni et al., 2017; Iglesias et al., 2018) and transport (Shi et al., 2015), abscisic acid (ABA)-mediated stomatal closure (Albertos et al., 2015; Wang et al., 2015), nitrogen assimilation (Frungillo et al., 2014), and nodule development (Matamoros et al., 2020).

To investigate the roles of GSNOR1 in tetraploid *N. tabacum*, the *NtGSNOR1a* and *NtGSNOR1b* knockout plants were generated *via* clustered regularly interspaced short palindromic repeats (CRISPR)/caspase 9 (Cas9) technology. Unexpectedly, spontaneous cell death was observed in the *NtGSNOR1a/1b* knockout plants under natural growth conditions, which has not been reported for the *Arabidopsis gsnor1* mutant previously. This spontaneous cell death phenotype was not rescued by knocking out *NtEDS1a/1b* and thus was SA-independent. To our surprise, the *NtGSNOR1a/1b* knockout plants displayed increased resistance to paraquat (1,1′-dimethyl-4,4′-bipyridinium dichloride)-induced cell death compared to wild-type (WT) plants, indicating that GSNOR1 in *Nicotiana* species functions as both positive and negative regulator of cell death under different cellular conditions. Interestingly, whereas the resistance to *Tobacco mosaic virus* (TMV) was enhanced in the *NtGSNOR1a/1b* knockout plants, the resistance to *Pst* DC3000 was significantly reduced (*p* < 0.001), indicating that *NtGSNOR1a/1b* have opposite effects on viral and bacterial pathogen infections in *N. tabacum*.

## Results

### Generation of the CRISPR/Cas9 Lines That Simultaneously Knock Out Two Alleles of *NtGSNOR1a* and Two Alleles of *NtGSNOR1b* in Tetraploid *N. tabacum*

**Figure 1:**
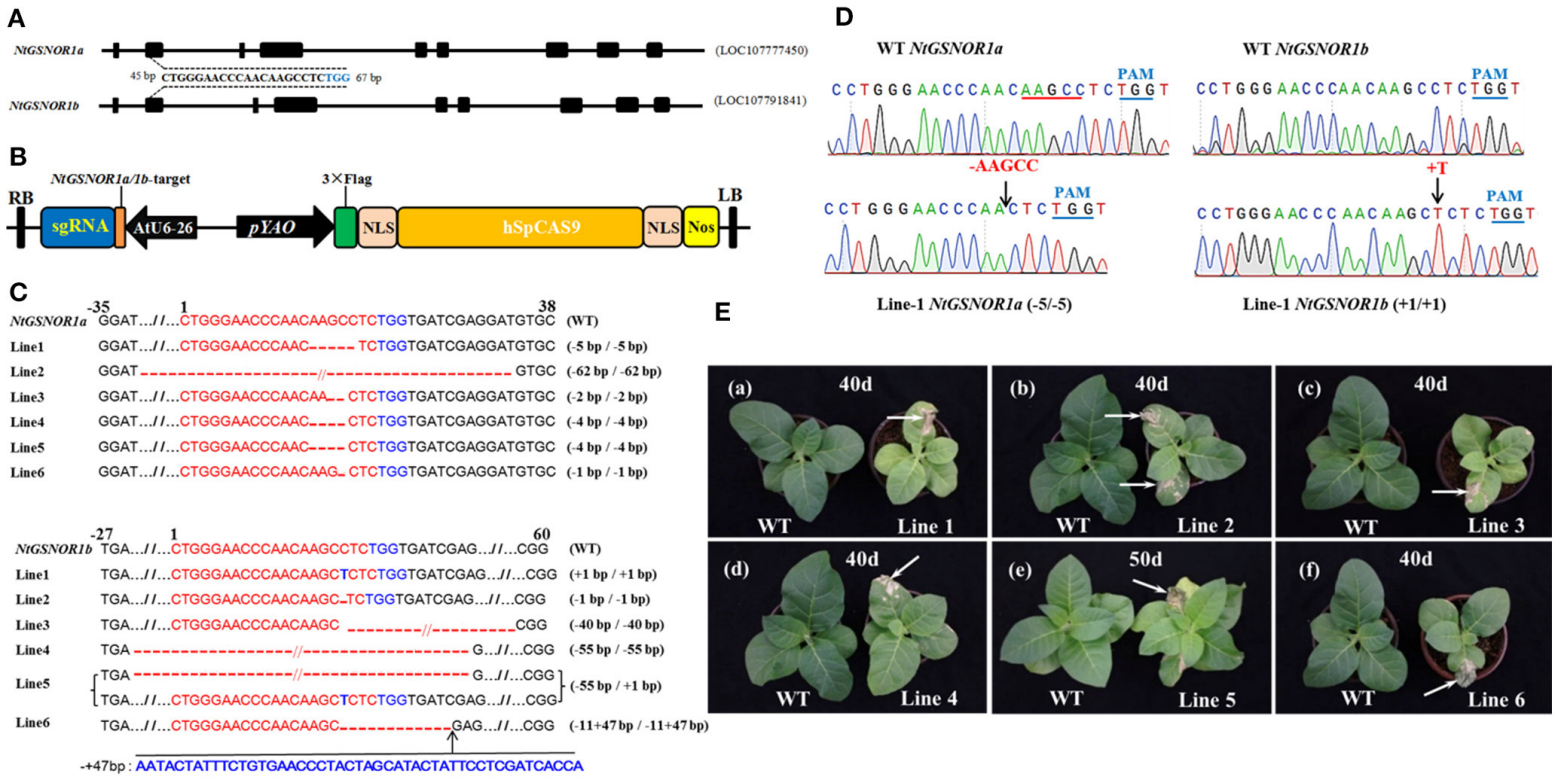
Knocking out *NtGSNOR1a* and *NtGSNOR1b* simultaneously results in spontaneous cell death on the leaves of *N. tabacum*. **(A)** Gene model of *NtGSNOR1a* (LOC107777450) and *NtGSNOR1b* (LOC107791841). A consensus sequence between *NtGSNOR1a* and *NtGSNOR1b* at the second exon (45–65 bp downstream of start codon ATG of the open reading frame) was used as a guide to target both genes simultaneously. The TGG in blue represents PAM sequence. **(B)** The diagram of the CRISPR/Cas9 construct used for generating transgenic plants. **(C)** The sequence alignment of the six identified CRISPR/Cas9 knockout lines. The short horizontal lines in red represent the deletions; the TGG in blue represents PAM sequence; and the red letters represent the insertions; // represent some bases were omitted for the purpose of saving space. The mutation types of these six CRISPR/Cas9 lines were shown by the labeling on the right. **(D)** Comparison of the sequencing chromatograms between the wild type (WT) and the CRISPR/Cas9 knockout line 1 at the edited region. Arrows pointed to the sites of deletions and insertions. + represents insertion, and - represents deletion. The PAMs are marked in blue letters and lines. **(E)** The spontaneous cell death phenotype is displayed on the leaves of all these six CRISPR/Cas9 lines. Arrows pointed to the regions with cell death.

It has been known that NO promotes an increase in ROS-induced cell death (Delledonne et al., 1998; Durner et al., 1998). However, the mechanism behind this NO-promoted cell death is not understood. To investigate the roles of GSNOR1 in cell death and disease resistance in tetraploid *N. tabacum*, we designed to simultaneously knockout four alleles of two highly related orthologs of *Arabidopsis GSNOR1* gene, namely, *NtGSNOR1a* (LOC107777450) and *NtGSNOR1b* (LOC107791841) and created *NtGSNOR1a/NtGSNOR1b* double knockout lines in *N. tabacum* cv. Samsun, which carries TMV-resistant gene, *N*, using CRISPR/Cas9 technology. The *NtGSNOR1a* and *NtGSNOR1b* share overall 98% identity at the nucleotide level. A consensus 20-bp sequence shared between *NtGSNOR1a* and *NtGSNOR1b* within the second exon of *NtGSNOR1a/1b* was used as a guide to target both genes (Figures 1A,B). The blast searching using the target sequence against the tobacco genome revealed that the target sequence only specifically matched to the *NtGSNOR1a/1b* target sites but not the other sites within the tobacco genome, with the closest match displayed 4-nt mismatches, which potentially could rule out the possibility of off-target effect. Multiple T0 transgenic lines were generated through *Agrobacterium*-mediated transformation, and six independent CRISPR/Cas9 lines, in which two alleles of *NtGSNOR1a* and two alleles of *NtGSNOR1b*, respectively, were simultaneously knocked out, were identified in the T0, T1, or T2 populations. The types of mutations for each of the four alleles in these six CRISPR/Cas9 lines were summarized in Figure 1C. The sequencing chromatograms showing the insertions and deletions (InDels) in the sequences of *NtGSNOR1a* and *NtGSNOR1b* of the CRISPR/Cas9 lines 1 were presented in Figure 1D. The sequencing chromatograms of the CRISPR/Cas9 line 2 to line 6 were presented in Supplementary Figures 1–5. Translational analysis indicated that the mutations (InDels) within all four alleles of *NtGSNOR1a/1b* in these six CRISPR/Cas9 lines could result in premature stop codons and would generate truncated proteins. Thus, these CRISPR/Cas9 lines can be considered independent knockout lines of both *NtGSNOR1a* and *NtGSNOR1b* (*NtGSNOR1a/1b*).

### The Spontaneous Cell Death Occurs on the Leaves of the *NtGSNOR1a/1b* Knockout Plants

**Figure 2:**
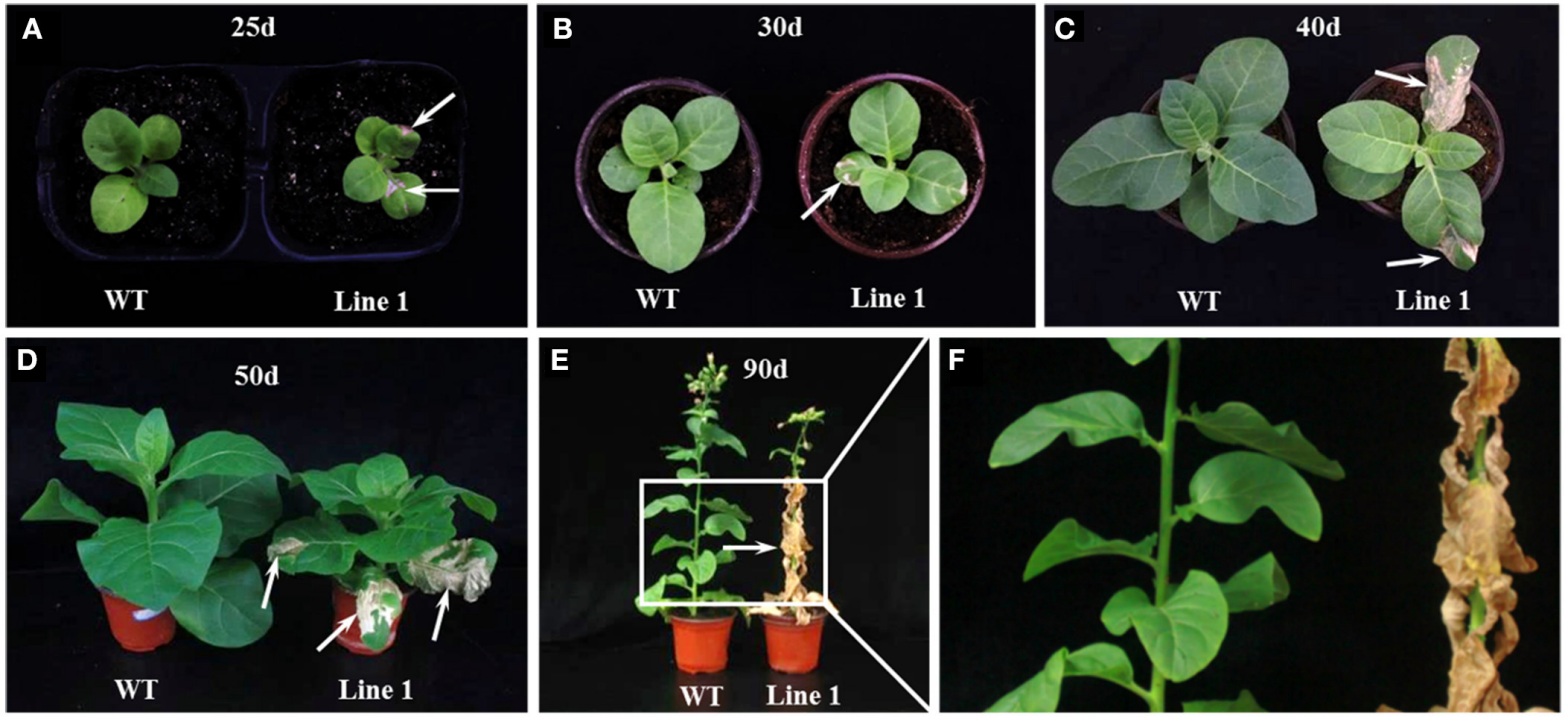
The spontaneous cell death phenotype of *NtGSNOR1a/1b* knockout plants at different developmental stages. **(A)** The spontaneous cell death phenotype could occur as early as 25 days post germination. **(B–F)** The cell death was aggravated with the development and progressed to upper systemic leaves. **(D–F)** Almost all the leaves of the knockout lines eventually died at 90 days post germination. **(E,F)** The enlarged image in the rectangle area in **(E)** was shown in **(F)**. Arrows pointed to the dead cells.

These six CRISPR/Cas9 knockout lines shared a similar morphological phenotype with a smaller overall stature compared to the WT plants through the entire life cycle (Figures 1E, 2). To our surprise, a severe spontaneous cell death phenotype was observed on the leaves of these *NtGSNOR1a/1b* knockout lines (Figure 1E), which was not observed in the WT plants throughout the entire growth season. The spontaneous cell death was not synchronous even for the same batch of the knockout plants. The cell death showed up earlier on some plants than on the others. The spontaneous cell death was mainly on the fully expanded true leaves independent of developmental stage. It could occur as early as the two-leaf stage (Figure 2A). It usually appeared on the lower leaves and spread progressively to the upper systemic leaves (Figures 1B–E). The cell death was similar to HR cell death, which initially displayed as water-soaked spots and eventually dried out. These results indicate that *Nt*GSNOR1 is a negative regulator of cell death.

### Both NO and H_2_O_2_ Were Overaccumulated in the *NtGSNOR1a/1b* Knockout Plants

**Figure 3:**
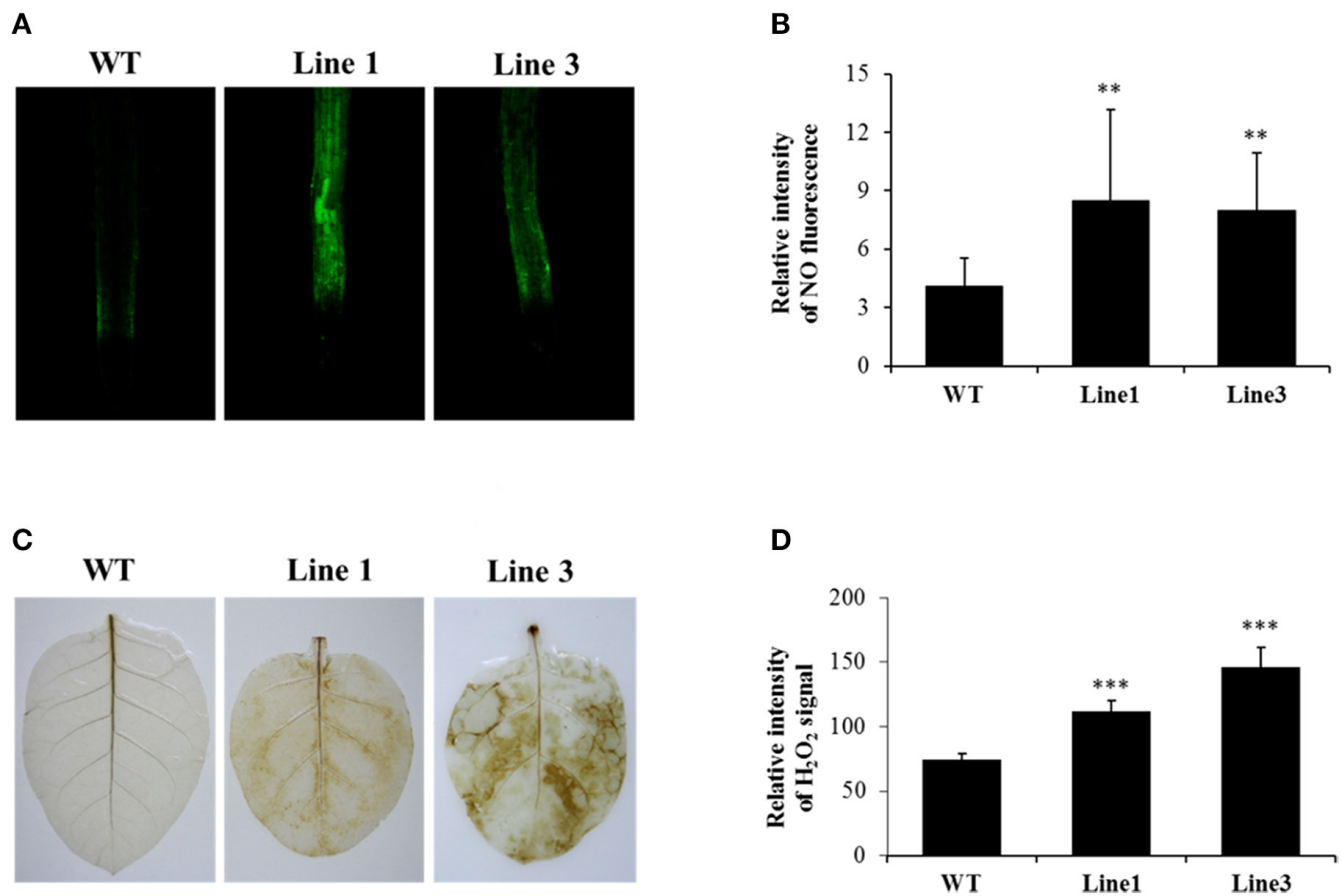
Knocking out *NtGSNOR1a/1b* leads to enhanced accumulation of both NO and H_2_O_2_. **(A)** NO level in the roots of 15-day-old plants was visualized by staining using 4,5-diaminoflorescein diacetate according to He et al. (2004). **(B)** The fluorescence intensity shown in **(A)** was quantified by ImageJ. **(C)** H_2_O_2_ was detected by staining the leaves of 40-day-old plants with 3,3'-diaminobenzidine (DAB) as described in section Materials and Methods. Oxidized DAB formed a reddish-brown deposit (examples of these deposits are indicated by the white arrows). **(D)** The intensity of the reddish-brown deposit shown in **(C)** was quantified by ImageJ. ** and *** indicate significant differences at 0.01 and 0.001 levels, respectively, by the Student's *t*-test.

To investigate the effect of *NtGSNOR1a/1b* knockout on NO and H_2_O_2_ accumulation in tobacco, we compared the NO and H_2_O_2_ levels between WT and the CRISPR/Cas9 lines using 4,5-diaminoflorescein diacetate and 3,3′-diaminobenzidine (DAB) staining, respectively. Consistent with the results observed for *Arabidopsis hot5-2*/*gsnor1-3/par2-1* mutants, NO level was significantly higher (*p* < 0.001) in the roots of the *NtGSNOR1a/1b* knockout plants of two independent lines compared with that of the WT plants (Figures 3A,B). Contrary to the results observed for *gsnor1-3* mutant in *Arabidopsis*, H_2_O_2_ level on the leaves of the *NtGSNOR1a/1b* knockout lines showing the sign of cell death was also higher than those of WT plants (Figures 3C,D), although little or no H_2_O_2_ was detectable on the leaves of the *NtGSNOR1a/1b* knockout lines without showing any cell death. Together, these results indicate that NO and H_2_O_2_ were overaccumulated in the *NtGSNOR1a/1b* knockout plants and the spontaneous cell death that occurred on the *NtGSNOR1a/1b* knockout plants could result from synergistic interactions between the overaccumulated NO and H_2_O_2_.

### The Spontaneous Cell Death on the Leaves of the *NtGSNOR1a/1b* Knockout Plants Is Not Dependent on the SA Pathway

**Figure 4:**
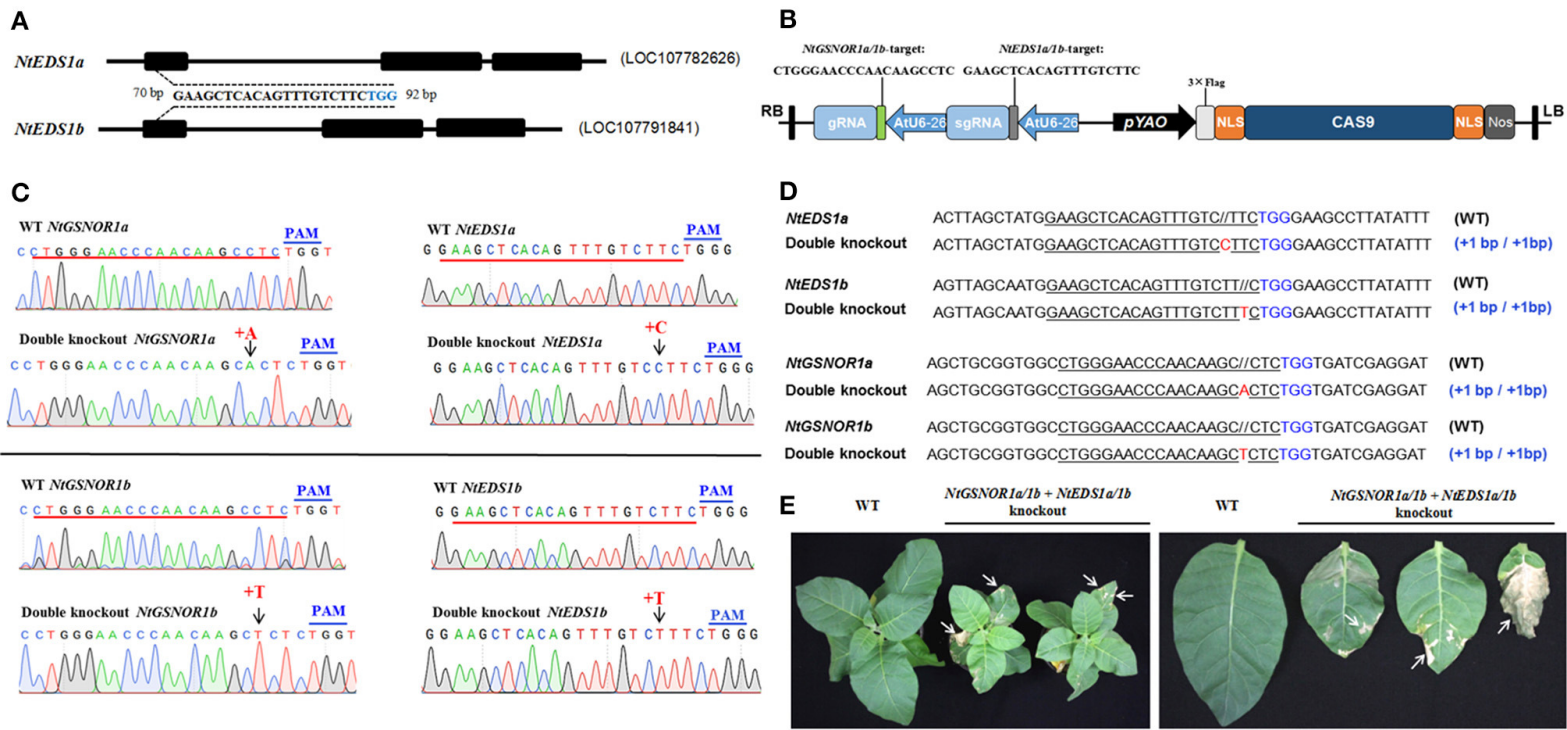
Knocking out *NtEDS1a/1b* cannot reverse the spontaneous cell death phenotype observed in *NtGSNOR1a/1b* knockout lines. **(A)** Gene model of *NtEDS1a* (LOC107782626) and *NtEDS1b* (LOC107791841). A consensus sequence between *NtEDS1a* and *NtEDS1b* at the first exon (70–92 bp downstream of start codon ATG of the open reading frame) was used as a guide to target both genes simultaneously. The TGG in blue represents PAM sequence. **(B)** The diagram of the CRISPR/Cas9 construct used for generating transgenic plants that knocks out both *NtEDS1a/1b* and *NtGSNOR1a/1b*. **(C)** Comparison of the sequencing chromatograms near the edited regions between the wild type (WT) and the CRISPR/Cas9 line, in which both *NtEDS1a/1b* and *NtGSNOR1a/1b* are knocked out simultaneously. Arrows pointed to the sites of insertions. + represents insertion. The PAM sequences are marked in blue. **(D)** The sequence alignments of the identified CRISPR/Cas9 double knockout line at both the *NtEDS1a/1b* and *NtGSNOR1a/1b* loci. The short horizontal lines in red represent the deletions; the TGG in blue represents PAM sequence; and the red letters represent the insertions; // represents extra space added for the purpose of the perfect sequence alignment. The mutation types of the CRISPR/Cas9 line were presented by the labeling on the right. **(E)** The spontaneous cell death phenotype is displayed on the leaves of the CRISPR/Cas9 double knockout line, in which both *NtEDS1a/1b* and *NtGSNOR1a/1b* are knocked out simultaneously. Arrows pointed to the regions with cell death.

EDS1 is required for SA accumulation (Rustérucci et al., 2001), and the constitutive cell death of many autoimmune mutants is dependent on EDS1 (Zhou and Zhang, 2020). To examine whether the spontaneous cell death on the *NtGSNOR1a/1b* knockout plants is SA-dependent, we knocked out four alleles of *NtEDS1a/1b* and four alleles of *NtGSNOR1a/1b* simultaneously in *N. Samsun* (NN) using the CRISPR/Cas9 construct carrying two guides (Figure 4A). The same 20-bp guide was used for targeting *NtGSNOR1a/1b* (Figure 1A), while a 20-bp identical sequence shared between *NtEDS1a* and *NtEDS1b* was used for targeting *NtEDS1a/1b* (Figure 4B). In the T1 or T2 populations, a CRISPR/Cas9 line that simultaneously knocked out four alleles of *NtGSNOR1a/1b* as well as four alleles of *NtEDS1a/1b* was identified. A single-base homozygous insertion was presented in both *NtGSNOR1a/1b* and *NtEDS1a/1b* alleles of this double knockout line (Figures 4C,D). A single “A” homozygous insertion was presented 4 bp upstream of PAM (TGG) for the two alleles of *NtGSNOR1a*. A single “T” homozygous insertion was presented 4 bp upstream of PAM for the two alleles of *NtGSNOR1b*. A single “C” homozygous insertion was presented 4 bp upstream of PAM for the two alleles of *NtEDS1a*. A single “T” homozygous insertion was presented 4 bp upstream of PAM for the two alleles of *NtEDS1b* (Figures 4C,D). Again, the single-base insertion in all these alleles of both the *NtGSNOR1a/1b* and the *NtEDS1a/1b* could lead to premature stop codons and would generate truncated proteins. Thus, both *NtGSNOR1a/1b* (four alleles) and *NtEDS1a/1b* (four alleles) were simultaneously knocked out in this CRISPR/Cas9 line. These results reveal the power of CRISPR/Cas9 technology in resolving gene redundancy in polyploidy plant species.

To further confirm that the *NtEDS1a/1b* are indeed knocked out in the *NtGSNOR1a/1b/NtEDS1a/1b* double knockout lines, we transiently overexpressed *GmMEKK1 via* agro-infiltration on the leaves of the double knockout line, which can trigger SA-dependent cell death (Xu et al., 2018). As expected, the HR cell death induced by overexpressing *GmMEKK1* was observed on the leaves of the *NtGSNOR1a/1b* knockout plants but not on the leaves of the *NtGSNOR1a/1b/NtEDS1a/1b* double knockout plants (Supplementary Figure 6), indicating that the *NtEDS1a/1b* is indeed knocked out in the double knockout lines. The fact that the spontaneous cell death was still observed on the *NtGSNOR1a/1b/NtEDS1a/1b* knockout plants (Figure 4E) indicated that the spontaneous cell death of *NtGSNOR1a/1b* knockout plants is independent of the SA pathway.

### The *NtGSNOR1a/1b* Knockout Plants Are More Resistant to Paraquat-Induced Cell Death

**Figure 5:**
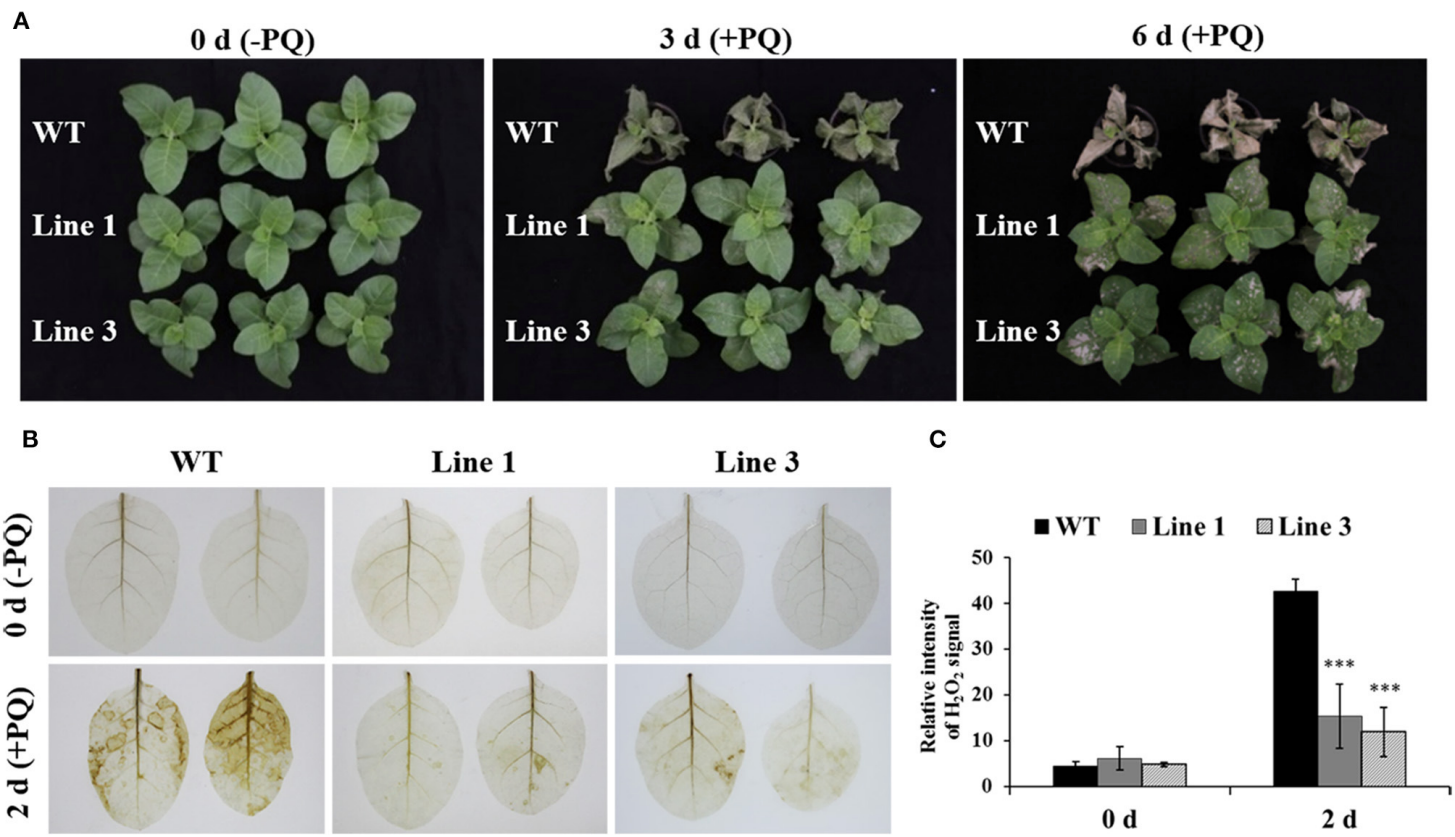
The *NtGSNOR1a/1b* knockout plants exhibited strong resistance to paraquat (PQ)-induced cell death through reducing PQ-induced H_2_O_2_ accumulation. **(A)** Here, 0.25% PQ was sprayed on the leaves of 40-day-old wild type (WT) and the CRISPR/Cas9 knockout lines 1 and 3. The images were taken at 0, 3, and 6 days post spraying. **(B)** The H_2_O_2_ accumulation on the leaves of WT and *NtGSNOR1a/1b* knockout plants at 0 and 2 days post-paraquat spraying visualized by DAB staining. **(C)** The intensity of the reddish-brown deposit shown in **(B)** was quantified by ImageJ. ** and *** indicate significant differences of *p*-value at 0.01 and 0.001, respectively, by the Student's *t*-test.

Paraquat, a non-selective herbicide, can induce cell death in plants (Dodge, 1971; Suntres, 2002). To examine whether knocking out *NtGSNOR1a/1b* in *N. tabacum* has a similar paraquat-resistant phenotype observed in *Arabidopsis* (Chen et al., 2009), 0.25% paraquat was sprayed onto the leaves of both the WT and the *NtGSNOR1a/1b* knockout plants before showing spontaneous cell death. While the WT plants almost died at 3 days post spraying (dps), two different *NtGSNOR1a/1b* knockout lines were still alive and relatively healthy (Figure 5A), indicating that the function of GNSOR1 on paraquat-induced cell death is conserved across plant species.

To investigate the resistant mechanism of the *NtGSNOR1a/1b* knockout plants to the paraquat-induced cell death, we visualized the H_2_O_2_ accumulation on the leaves of both WT and the *NtGSNOR1a/1b* knockout plants after paraquat spraying. Consistent with the result in *Arabidopsis* (Chen et al., 2009), we found that the level of H_2_O_2_ accumulation was much lower on the leaves of the *NtGSNOR1a/1b* knockout plants than that of the WT plants (Figures 5B,C), indicating that reduced level of paraquat-induced H_2_O_2_ production or accumulation could be at least one of the molecular mechanisms behind the resistance of the *NtGSNOR1a/1b* knockout plants to paraquat-induced cell death.

### Knocking Out *NtGSNOR1a/1b* Has Opposite Effects on *Tobacco mosaic virus* and *P. syringae* pv. *tomato* DC3000 Infections

**Figure 6:**
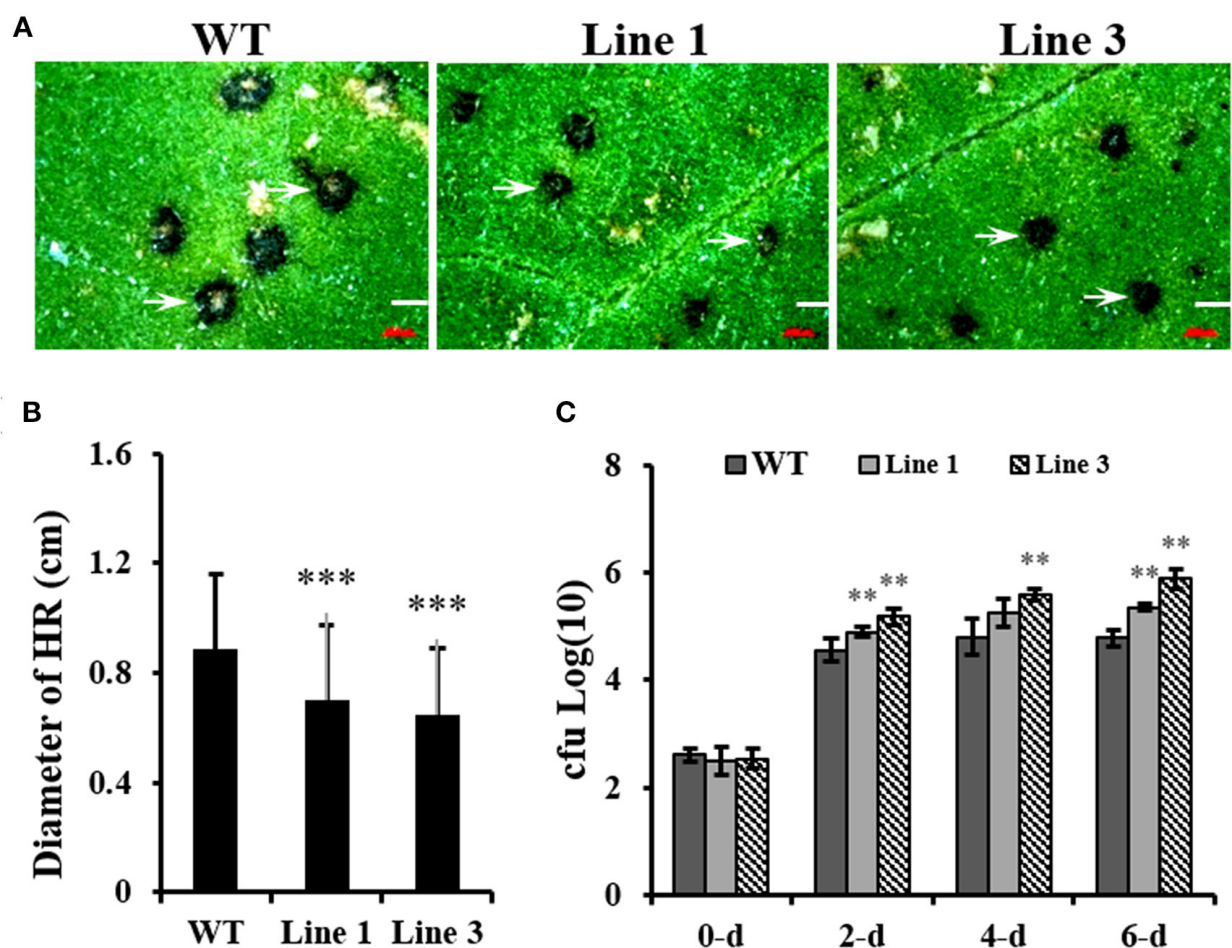
Knocking out *NtGSNOR1a/1b in N. tabacum* results in the enhanced *N* gene mediated resistance against TMV but reduced resistance against *Pseudomonas syringae* pv. *tomato* DC3000 (*Pst* DC3000). **(A)** The hypersensitive response (HR) lesions formed on the leaves of the 40-day-old *NtGSNOR1a/1b* knockout lines (Lines 1 and 3) are reduced in comparison to on that of wild-type (WT) *Samsun* (NN) plants in response to *Tobacco mosaic virus* (TMV) infection. **(B)** The sizes of HR lesions shown in **(A)** were measured under a dissecting microscopy. The lesions on the leaves of three independent plants of each line were measured. Over 30 lesions were measured on each infected leaf. *** represents significant difference at 0.001 level by the Student's *t*-test. **(C)** *NtGSNOR1a/1b* knockout plants support greater growth of *Pst* DC3000 compared to WT *Samsun* (NN) plants. The leaves of 40-day-old plants were infiltrated with *Pst* DC3000 (OD600 = 0.00001). The numbers of *Pst* DC3000 were counted at 0-, 2-, 4-, and 6-day post-inoculation and expressed as log10. ** represents significance at *p*-value 0.01 by the Student's *t*-test.

Contradictory results were obtained regarding the role of *Arabidopsis* GSNOR1 in disease resistance using *gsnor1-3* null mutant (Feechan et al., 2005) and GSNOR1 antisense lines (Rustérucci et al., 2007). However, the role of GSNOR1 in other plant species has not been investigated. To test the effect of *NtGSNOR1a/1b* knockout on disease resistance in *Nicotiana* species, we inoculated TMV on both WT *N. tabacum Samsun* (NN) and the *NtGSNOR1a/1b* knockout plants. It was found that the sizes of HR lesions triggered by TMV infection on the leaves of the *NtGSNOR1a/1b* knockout plants were significantly smaller than those on the leaves of *N. tabacum* cv. Samsun (NN) at 5 days post inoculation (dpi) (Figures 6A,B). Appearance of local HR lesions reflects both replication and cell-to-cell movement of the virus (Meshi et al., 1982). Consistent with this statement, we showed that the transcript level of TMV capsid protein (CP) was much lower on the knockout leaves than on the WT leaves (Supplementary Figure 7). However, we cannot exclude a possibility that the smaller sizes of the HR on the leaves of the knockout plants could also reflect a decreased ability for N protein to trigger the HR upon TMV infection.

To examine the effect of knocking out *NtGSNOR1a/1b* on bacterial infection, the upper leaves of both WT and *NtGSNOR1a/1b* knockout plants without spontaneous cell death were infiltrated with *Pst* DC3000 as the cell death on the lower leaves interferes with the bacterial growth assay. As shown in Figure 6C, *Pst* DC3000 multiply better on the leaves of the *NtGSNOR1a/1b* knockout plants than on the leaves of the WT plants (Figure 6C), indicating that *Nt*GSNOR1a/1b positively regulates the basal resistance to a bacterial pathogen. Together, these results indicate that knocking out *NtGSNOR1a/1b* has opposite effects on resistance to different types of pathogens with different infecting strategies.

## Discussion

In this report, we found that both anti- and pro-cell death effects were observed in the *NtGSNOR1a/1b* knockout plants, reinforcing the notion that NO can play either anti- or pro-death roles depending on cellular conditions (Wang et al., 2013; Wendehenne et al., 2014) and the altered NO homeostasis plays key roles in switching its functions during the HR. In addition, we found that knocking out *NtGSNOR1a/1b* has opposite effects on viral and bacterial resistance.

The cell death phenotype of the *NtGSNOR1a/1b* knockout plants under natural conditions was not reported for the *gsnor1-3/hot5/par2-1* mutants (Feechan et al., 2005; Lee et al., 2008; Chen et al., 2009) or antisense lines of *GSNOR1* in *Arabidopsis* (Rustérucci et al., 2007), although spontaneous cell death was observed on the leaves of *GSNOR1*-silenced tomato plants by virus-induced gene silencing (VIGS) (Liu et al., 2017) and an accelerated cell death was observed on the leaves of *gsnor1-3* mutant upon infections by avirulent bacterial strains or by an avirulent oomycete isolate (Rustérucci et al., 2007; Yun et al., 2011). The generation of both NO and H_2_O_2_ is simultaneously induced under many different stress conditions (Delledonne et al., 1998; Clarke et al., 2000; Lin et al., 2012; Scheler et al., 2013), indicating that they coexist in most cellular conditions. Both NO and H_2_O_2_ was significantly higher in the *NtGSNOR1a/1b* knockout plants compared to WT plants (Figure 3). The increased H_2_O_2_ level in the *NtGSNOR1a/1b* knockout plants could be induced by the overaccumulation of NO in these plants, since it has been reported previously that NO is essential for generating elicitor- or pathogen-inducible ROS and that ROS likely functions downstream of NO (Rasul et al., 2012; Wang L. et al., 2014; Kulik et al., 2015). It has been reported that efficient activation of hypersensitive cell death required a balance between NO and ROS production (Delledonne et al., 1998, 2001). NO/H_2_O_2_ ratio between 0.25 and 2 was effective in inducing cell death. NO/H_2_O_2_ ratio during pathogen induction of the HR or during induction of cell death by exogenous NO and artificial oxidative burst falls within this range (Delledonne et al., 2001). Based on this ratio model, we propose the following explanations for the naturally occurring cell death (Figures 1E, 2) and the reduced paraquat-induced cell death observed in the *NtGSNOR1a/1b* knockout plants (Figure 5A). Under normal growth conditions, NO level is high in the *NtGSNOR1a/1b* knockout plants, but H_2_O_2_ level is relatively low compared to the NO level. As a result, the NO/H_2_O_2_ ratio falls above the high end of the optimal ratio range so that cell death is not triggered. At certain development stages or under mild stress conditions, H_2_O_2_ is induced gradually. Once the H_2_O_2_ level is accumulated to a certain threshold level, the NO/H_2_O_2_ ratio falls within the optimal range, natural cell death occurs (Figures 1E, 2). In paraquat-sprayed leaves, tremendous amount of H_2_O_2_ is induced, causing the NO/H_2_O_2_ ratio to fall below the low end of the optimal ratio range and thus the cell death is prevented.

However, given the promiscuous natures of both NO and H_2_O_2_, the mechanism of NO- and H_2_O_2_-induced cell death could be multifaceted. Firstly, in plants, H_2_O_2_ and NO can chemically react to produce singlet oxygen or hydroxyl radicals, both of which have effects on causing cell death (Zaninotto et al., 2006). However, hydroxyl radical ONOO^−^, which can cause cell death in animals, is not toxic to plants (Delledonne et al., 2001). Rather, it is likely that the formation of ONOO^−^ can serve as a protective mechanism by scavenging ${\text{O}}_{2}^{-}$ from reactions causing cellular damage (Delledonne et al., 2001). Consistent with this statement, we found that the H_2_O_2_ level in the *NtGSNOR1a/1b* knockout plants was much lower than that in WT plants sprayed with paraquat (Figure 5B), suggesting that increased RNS accumulation can neutralize and/or detoxify the effects of ROS.

Given the role of GSNOR1 in maintaining homeostasis of cellular *S*-nitrosylation (Liu et al., 2001; Feechan et al., 2005), it is also possible that the naturally occurring cell death (Figure 1) and resistance to paraquat-induced cell death (Figure 5) in the *NtGSNOR1a/1b* knockout plants could be mediated by *S*-nitrosylation through modulating different target proteins. *S*-nitrosylation of proapoptotic and antiapoptotic regulators is a common mechanism to regulate apoptosis in animal systems (Mannick, 2007; Iyer et al., 2014). Stimulation or stabilization of antiapoptotic factors by *S*-nitrosylation inhibits cell death (Mannick, 2007; Iyer et al., 2014), whereas stimulation of proapoptotic factors by *S*-nitrosylation promotes or initiates apoptosis (Mannick, 2007; Iyer et al., 2014). In plants, it has been shown that NO increases H_2_O_2_ levels by inhibiting catalase and ascorbate peroxidase, two major ROS-scavenging enzymes, through *S*-nitrosylation (Lin et al., 2012; Ortega-Galisteo et al., 2012; de Pinto et al., 2013). It is possible that the increased accumulation of H_2_O_2_ (Figure 3C) and naturally occurring cell death observed in the *NtGSNOR1a/1b* knockout plants (Figure 1) are at least partially caused by inhibiting ROS detoxification through *S*-nitrosylation of both catalase and ascorbate peroxidase. On the other hand, *S*-nitrosylation of the *Arabidopsis* cytosolic APX1 at Cys^32^ enhances its enzymatic activity of scavenging H_2_O_2_, resulting in an increased resistance to oxidative stress (Yang et al., 2015). It also has been recently shown that *S*-nitrosylation of the NADPH oxidase, *At*RBOHD, at Cys^890^, abolishing its ability to synthesize ROS and thus functioning as a negative feedback loop limiting the HR (Yun et al., 2011), raises the possibility that the resistance to paraquat-induced cell death observed in the *NtGSNOR1a/1b* knockout plants is at least partially attributed to the feedback inhibition of H_2_O_2_ production by *At*RBOHD and APX1. We recently provide evidence that NO may trigger cell death in tomato (*Solanum lycopersicum*) by inhibiting the activity of phosphoinositide-dependent kinase 1 (*Sl*PDK1), a conserved negative regulator of cell death in yeasts, mammals, and plants, *via S*-nitrosylation (Liu et al., 2017). *Sl*PDK1 is primarily *S*-nitrosylated on Cys^128^ and substitution of Cys^128^ with serine completely abolished *Sl*PDK1 kinase activity, suggesting that *S*-nitrosylation of Cys^128^ is responsible for *Sl*PDK1 inhibition (Liu et al., 2017). These results establish a potential link between NO-triggered cell death and inhibition of the kinase activity of tomato PDK1. It is possible that the spontaneous cell death observed on the leaves of *NtGSNOR1a/1b* knockout plants is a consequence of inhibition of the *Nt*PDK1 activity.

Even though the naturally occurring spontaneous cell death is not observed in *Arabidopsis gsno1-3* plants, HR was accelerated in both *GSNOR1* antisense plants and in *atgsnor1-3* mutant plants upon infection by avirulent bacterial strains or by an avirulent oomycete isolate (Rustérucci et al., 2007; Yun et al., 2011), indicating a conserved negative role of GSNOR1 from different plant species in triggering cell death. Interestingly, accelerated cell death is independent of SA and H_2_O_2_, as the cell death in *gsnor1-3/sid2* and *gsnor1-3/atrbohD, gsnor1-3/atrboF* double mutants, and *gsnor1-3/atrbohD/atrbohF* triple mutants displays the same degree of cell death as in *gsnor1-3* plants, and H_2_O_2_ level is less in *gsnor1-3* plants than that in Col-0 plants (Yun et al., 2011). Consistent with this result, we found that the spontaneous cell death occurring on the *NtGSNOR1a/1b* knockout plants was not suppressed by knocking out the *NtEDS1a/1b* (Figure 4), strongly indicating that the spontaneous cell death is SA-independent.

The *NtGSNOR1a/1b* knockout plants displayed increased resistance to TMV but reduced resistance to *Pst* DC3000 (Figure 6). The opposite effects of silencing or knocking out *GSNOR1* on the resistance to different types of pathogens were also observed in *Arabidopsis* (Feechan et al., 2005; Rustérucci et al., 2007; Yun et al., 2011). Whereas, the basal resistance to *Pst* DC3000 and Noco2 race of the *H. parasitica*, RPM1- or RPS4-mediated R resistance to *Pst* DC3000 (*avrB*) and *Pst* DC3000 (*avrRps4*), and non-host resistance to wheat powdery mildew pathogen *Bgt* are all compromised in *Arabidopsis gsnor1-3* mutant (Feechan et al., 2005), an enhanced basal resistance to oomycete Pp Noco2 and an increased SAR were observed in *Arabidopsis GSNOR1* antisense plants (Rustérucci et al., 2007). Espunya et al. (2012) attributes these opposing phenotypes to the partial reduction (antisense line) vs. the complete loss of function (knockout mutant) in GSNOR1 activity. Interestingly, contrary to their own report that various types of resistance are compromised in *gsnor1-3* mutant plant (Feechan et al., 2005), Yun et al. (2011) recently showed that an enhanced resistance to *Hyaloperonospora arabidopsidis* isolate Emwa1, which is recognized by RPP4, was observed in *gsnor1-3* mutant in an SA- and H_2_O_2_-independent manner (Yun et al., 2011). This resistance seemed to be attributed sorely to increased SNO levels in *gsnor1-3* mutant (Yun et al., 2011). Thus, the opposing results in resistance to different types of pathogens observed in *gsnor1-3* mutant cannot be explained sorely by the degree of reduction in GSNOR1 activity. Rather, it reflects the complexity and hierarchy of NO signaling events (Wendehenne et al., 2014).

Multiple proteins involved in defense responses could be differentially or simultaneously *S*-nitrosylated in response to pathogen infections. Depending on the severity of nitrosative stress, the subcellular compartmentalization of both GSNO and its target proteins, GSNO could either promote or inhibit defense responses. As the functions of the positive regulators of SA-dependent pathway, NPR1, TGA1, and SBP3, and the function of a suppressor of negative regulator of defense responses, SRG1, are negatively regulated by *S*-nitrosylation (Tada et al., 2008; Wang et al., 2009; Lindermayr et al., 2010; Cui et al., 2018), it is reasonable to assume that the compromised resistance of the *NtGSNOR1a/1b* knockout plants to *Pst* DC3000 (Figure 6C) could also be combined results of compromised SA-mediated resistance and de-repression of immunity inhibition. However, Yun et al. (2011) reported that the cell death triggered by incompatible pathogens in *gsnor1-3* is SA- and H_2_O_2_-independent and the cell death triggered in *gsnor1-3* is enough to confer the *RPP4*-mediated resistance to *Hyaloperonospora arabidopsidis* isolate Emwa1, suggesting that the resistance is SA-independent (Yun et al., 2011). However, our results indicated that the spontaneous cell death on the *NtGSNOR1a/1b* knockout plants (Figures 1, 2) does not confer bacterial resistance (Figure 6B). Intriguingly, contrary to the response to bacterial infection, an enhanced resistance against TMV was observed in the *NtGSNOR1a/1b* knockout plants (Figure 6A). The increased resistance to TMV could be due to inhibitory effects of the overaccumulated Reactive nitrogen species (RNS) and ROS (Figure 3) on virus replication or movement or both. It is also possible that the viral proteins involved in replication or movement is inhibited by *S*-nitrosylation. Alternatively, the enhanced resistance of the *NtGNSOR1a/1b* knockout plant to TMV could also be due to the inhibition of negative regulators of defense responses by *S*-nitrosylation. For example, *Arabidopsis* SUMO E2 enzyme, SCE1, plays a negative role in immune responses. *S*-nitrosylation SCE1 at Cys^139^ inhibits its SUMO-conjugating activities, leading to immune activation by relieving SUMO1/2-mediated suppression (Skelly et al., 2019). Depending on the cell type, subcellular localization, developmental stages, and plant–pathogen interactions, as well as its diffuse and promiscuous nature, NO can exert differential or even opposite effects on cell death and immunity through both *S*-nitrosylation-dependent and -independent mechanisms, which might explain the opposite effects of *NtGSNOR1a/1b* knocking out on *Pst* DC3000 and TMV resistance.

The CRISPR/Cas9-mediated genome editing tool has been extensively applied in plant biotechnology since its development (Xie and Yang, 2013; Wang Y. et al., 2014; Wang et al., 2015; Char et al., 2017, 2020; Chen et al., 2019; Oliva et al., 2019). Our results strongly indicated that CRISPR/Cas9 is a powerful technology not only in resolving gene redundancy in tetraploid tobacco (Figures 1, 4) but also in studying the functional connections and/or interactions of different genes (Figure 4).

## Materials and Methods

### Plant Materials

*Nicotiana tabacum* cv. *Samsun*, carrying a TMV-resistant gene, *N*, was used in this study. The tobacco plants were maintained in the growth room at 22°C with a photoperiod of 16-h light/8-h dark.

### Agro-Infiltration

*Agrobacterium* infiltration was performed as described (Liu et al., 2005). *Agrobacterium* GV3101 strain carrying the *35S*::*GmMEKK1a* construct (Xu et al., 2018), which is known to induce HR, was infiltrated into the leaves of the 30–40-day-old *N. tabacum* plants as indicated. The *35S*::*GFP* construct was included as a negative control. The leaf was photographed at 2 days post infiltration. The experiment was repeated three times (at least three plants each time) with similar results.

### CRISPR/Cas9 Constructs and Plant Transformation

Oligos of 20 nucleotides were chosen from the second exon of *NtGSNOR1a/1b* (CTGGGAACCCAACAAGCCTC, 45–65 bp downstream of start codon ATG of the open reading frame, was used as a guide to target both genes simultaneously) and first exon of *NtEDS1a/1b* (GAAGCTCACAGTTTGTCTTC, 70–92 bp downstream of the start codon ATG) (Figures 1A, 4B). Blast analysis indicated these two guides only perfectly matched the target sequences of *NtGSNOR1a/1b* and *NtEDS1a/1b*, respectively, but not any other sequences in the *N. tabacum* genome. The closest sequences in the genome to both guides displayed at least 4-nt mismatches. Thus, the off-target effects could potentially be ruled out with great probability. Both the sense and antisense strands of the chosen oligos were synthesized with ATTG and AAAC that are compatible to *Bsa* I sticky ends attached to the sense and antisense strands, respectively. The sense and antisense strands of the synthesized oligos were mixed in TE buffer and annealed to each other by heating at 98°C for 5 min followed by cooling to room temperature. The annealed double-strand oligos were subsequently ligated into the intermediate AtU6-26-sgRNA-SK vector (Yan et al., 2015) predigested with *Bsa* I. The constructed plasmid was double digested with *Nhe* I and *Spe* I (*Nhe* I and *Spe* I are isocaudomers), and the fragment of 642 bp containing the guide sequence was ligated into the destination vector *pCAMBIA-1300-pYAO:Cas9* predigested with *Spe* I and treated with alkaline phosphatase (Yan et al., 2015). The authenticity of the final construct was confirmed by sequencing.

For double targets (*NtGSNOR1a/1b* + *NtEDS1a/1b*) construct, the *Nhe* I and *Spe* I digested fragment from the intermediate vector containing *NtEDS1a/1b* guide was ligated into the destination vector containing *NtGSNOR1a/1b* guide digested with *Spe* I. The authenticity of the final construct was confirmed by sequencing.

The oligos used for target guides are:

*NtGSNOR1a/1b-*F: 5′-***ATTG***CTGGGAACCCAACAAGCCTC-3′;
*NtGSNOR1a/1b-*R: 5′-***AAAC***GAGGCTTGTTGGGTTCCCAG-3′;
*NtEDS1a/1b-*F: 5′-***ATTG***GAAGCTCACAGTTTGTCTTC-3′;
*NtEDS1a/1b-*R: 5′-***AAAC***GAAGACAAACTGTGAGCTTC-3′.

The CRISPR/Cas9 constructs were transformed into *N. tabacum* cv. *Samsun (NN)* as described previously (Horsch et al., 1985). Briefly, fully expanded leaves from 3-week-old tobacco plants were surface-sterilized with 70% ethanol and 0.5–2% NaClO_3_, respectively. Leaf discs of 0.5 cm in diameter were precultivated in MS medium for 3 days followed by incubating in Agrobacterium (GV3101) suspension solution for 20–30 min. Agrobacterium-infected leaf discs were cultivated in MS medium containing 1 mg/L Indole-3-acetic acid (IAA), 1 mg/L 6-BA in the growth room for 2 days, and then transferred to MS medium supplemented with 1 mg/L IAA, 1 mg/L 6-BA, 50 mg/L Hygromycin, and 25 mg/L rifampicin. Shoots were transferred to rooting medium containing IAA (1 mg/L), 50 mg/L Hygromycin, and 25 mg/L Timentin. The rooted transformants were finally transferred to soil and grown in a growth room.

### Screening of the Homozygous CRISPR/Cas9 Lines

The transgenic CRISPR/Cas9 plants generated were characterized by genomic PCR with specific primers listed below and followed by DNA sequencing. DNA was extracted from leaf tissue with the DNA extraction kit (Invitrogen). Sequence results were aligned against the wild type sequence to identify insertions and deletions (InDels). The sequencing chromatograms contained no double peaks but contained InDels indicating homozygous mutations. The double peaks in the chromatograms indicate a heterozygous insertion/deletion or WT/InDels. In this case, the genomic PCR products were cloned into the PMD19-T vector (Takara), and multiple clones were subjected to sequencing to determine the exact nature of InDels. The primers used for the genomic PCR or for screening positive clones by colony PCR are:

*NtGSNOR1a/1b-*F: 5′-CACTACTAGTATACCACTTA-3′
*NtGSNOR1a-*R: 5′-GTGGTTAACTTTATGAATAATTTGC-3′
*NtGSNOR1b-*R: 5′-TTAGAAACTCACATAGCCGG-3′
*NtEDS1a-*F: 5′-CCATCTTTGAGCTGCTAGCA-3′
*NtEDS1a-*R: 5′-GGGAACATATTCTTGGACATG-3′
*NtEDS1b-*F: 5′-CCATCTTTGAGCTGCTAAGT-3′
*NtEDS1b-*R: 5′-GGAATTAAGCTGATCAAACTA-3′

### *Tobacco mosaic virus* Infection and *P. syringae* pv. Tomato DC3000 Growth Assay

The properly diluted sap extracted from the TMV (U1 strain) infected leaves was rub-inoculated on the upper surface of the *N. tabacum* cv. *Samsun (NN)* leaves, as described previously (Whitham et al., 1994). The HR normally is visible at 2 dpi, and the photos were taken at 4 dpi. The primers used for qRT-PCR quantification of the TMV CP are:

*CP*-F: GCTCTCGAAAGAGCTCCGAT
*CP*-R: TTTATCGCGCTCCTTATGGC

The primers for endogenous reference gene *NtActin* are:

*NtActin*-F: TGGCATCACACTTTCTACAA
*NtActin*-R: CCACTGAGCACAATGTT

The *Pst* DC3000 of OD_600_ = 0.00001 was infiltrated into the leaves of respective tobacco lines, as described previously (Qi et al., 2018). The leaf discs of 0.5 cm in diameter were punched from the infiltrated areas and grounded in the 10 mM MgSO_4_ buffer. The saps were diluted 10, 100, and 1,000 times and spread on KB agar plates. Colony-forming unit (cfu) was obtained by counting the number of the colonies on a serial of plates.

### H_2_O_2_ Detection by 3,3′-Diaminobenzidine Staining

H_2_O_2_ was detected by an endogenous peroxidase-dependent *in situ* histochemical staining procedure using DAB (Sigma-Aldrich; Ren et al., 2002). Leaves of 30–40-day-old plants were detached and placed in a solution containing 1 mg/ml DAB (pH 5.5) for 2 h. The leaves were cleared by boiling in ethanol (96%) for 10 min and then stored in 96% ethanol. H_2_O_2_ production was visualized as a reddish-brown precipitate in cleared leaves (Thordal-Christensen et al., 1997; Ren et al., 2002).

### NO Detection by DAF-2DA Staining

NO detection was performed by DAF-FM DA staining as described (He et al., 2004). Briefly, leaf discs and 15-day-old seedlings were preincubated for 2 h at room temperature in a solution of 0.1 mM CaCl_2_, 10 mM KCl, 10 mM MES-Tris, pH 5, and then stained with 10 μM DAF-FM DA for 45 min. After rinsing with water, the sample was observed and photographed with an inverted Axiophot microscope (Zeiss) equipped with a digital camera (Diagnostic Instruments).

### Accession Numbers

The accession numbers for *NtGSNOR1a, NtGSNOR1b, NtEDS1a*, and *NtEDS1b* are LOC107777450, LOC107791841, LOC107782626, and LOC107791841, respectively.

### Statistics

Statistics were performed using the Student's *t*-test. ^**^ and ^***^ represent the significant differences between the WT control and the knockout plants at levels of 0.01 and 0.001, respectively.

## Data Availability Statement

The raw data supporting the conclusions of this article will be made available by the authors, without undue reservation.

## Author Contributions

J-ZL designed the experiments. Z-CL, Q-WR, JR, YG, X-TR, N-NW, H-YX, and XL performed the experiments. J-ZL wrote the manuscript with Z-CL's input. All authors contributed to the article and approved the submitted version.

## Conflict of Interest

The authors declare that the research was conducted in the absence of any commercial or financial relationships that could be construed as a potential conflict of interest.
