# Supplementary material for: Dual Roles of GSNOR1 in Cell Death and Immunity in Tetraploid Nicotiana tabacum
Source: Front Plant Sci. 2021 Feb 10;12:596234. doi: 10.3389/fpls.2021.596234 (PMC7902495; doi:10.3389/fpls.2021.596234)
Supplement: Supplementary file 1 [file Data_Sheet_1.PDF]

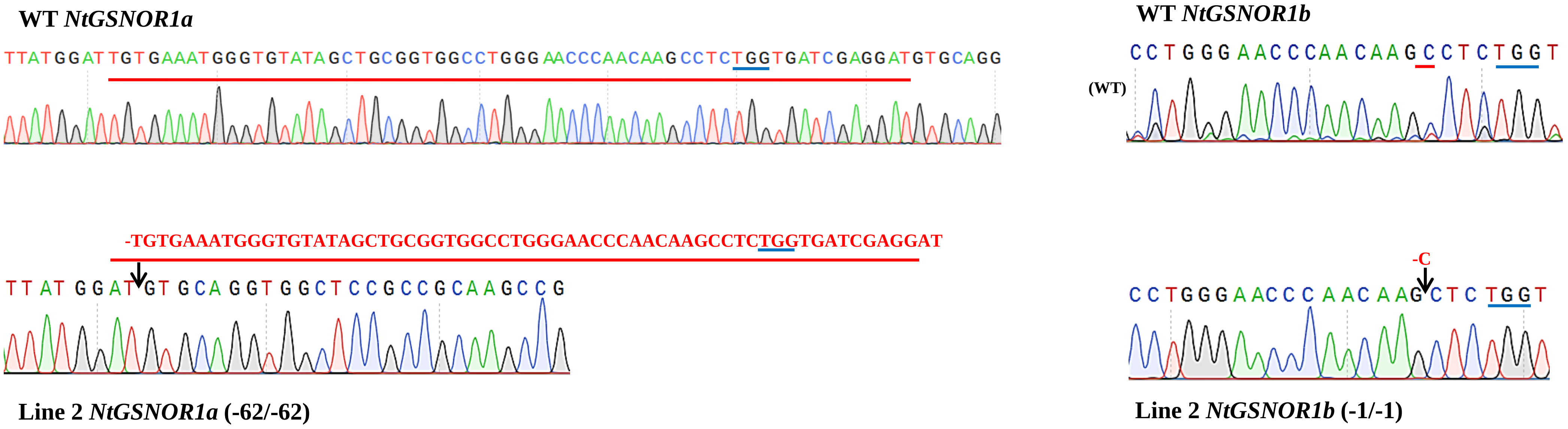

Fig. S1. Comparison of the sequencing chromatograms of *NtGSNOR1a* and *NtGSNOR1b* alleles between wildtype (top panel) and the transgenic CRISPR/CAS9 line 2 (bottom panel). The sequencing results indicated that a 62-bp sequence was deleted in the two alleles of *NtGSNOR1a* (bottom left) and a C was deleted in the two alleles of *NtGSNOR1b* (bottom right) of the CRISPR/CAS9 line 2. The positions of the deletions are pointed by arrows and the deleted sequences are marked by red underlines in the WT chromatograms and provided on top of the chromatograms of the CRISPR/CAS9 line 2. The PAM sequence is underlined in blue.

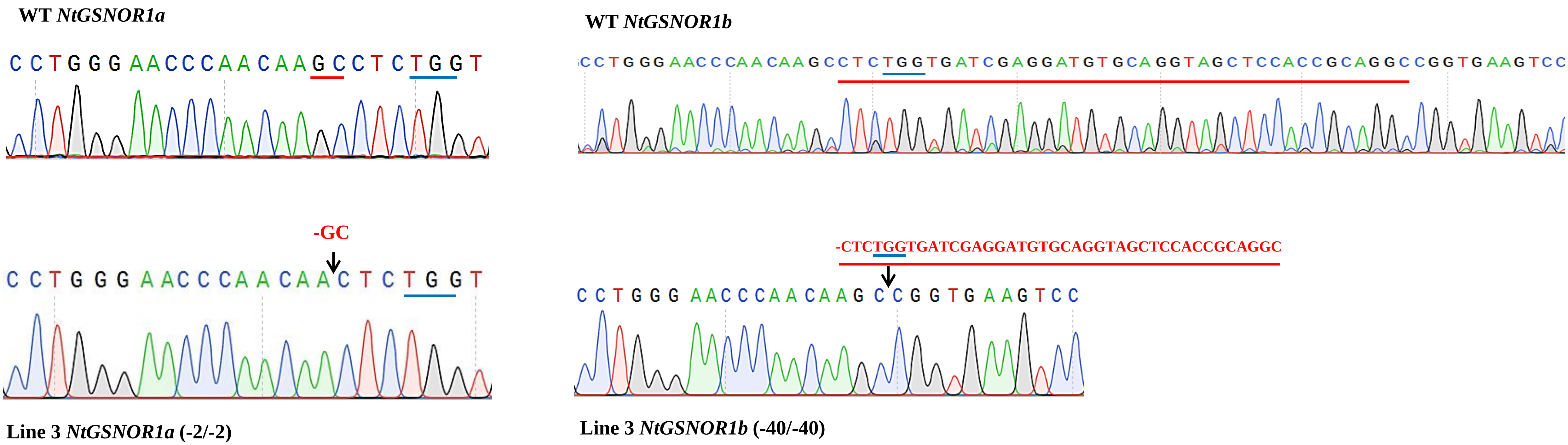

Fig. S2. Comparison of the sequencing chromatograms of *NtGSNOR1a* and *NtGSNOR1b* alleles between wildtype (top panel) and the transgenic CRISPR/CAS9 line 3 (bottom panel). The sequencing results indicated that a 2-bp sequence (GC) was deleted in the two alleles of *NtGSNOR1a* (bottom left) and a 40-bp sequence was deleted in the two alleles of *NtGSNOR1b* (bottom right) of the CRISPR/CAS9 line 3. The positions of the deletions are pointed by arrows and the deleted sequences are marked by red underlines in the WT chromatograms and provided on top of the chromatograms of the CRISPR/CAS9 line 3. The PAM sequence is underlined in blue.

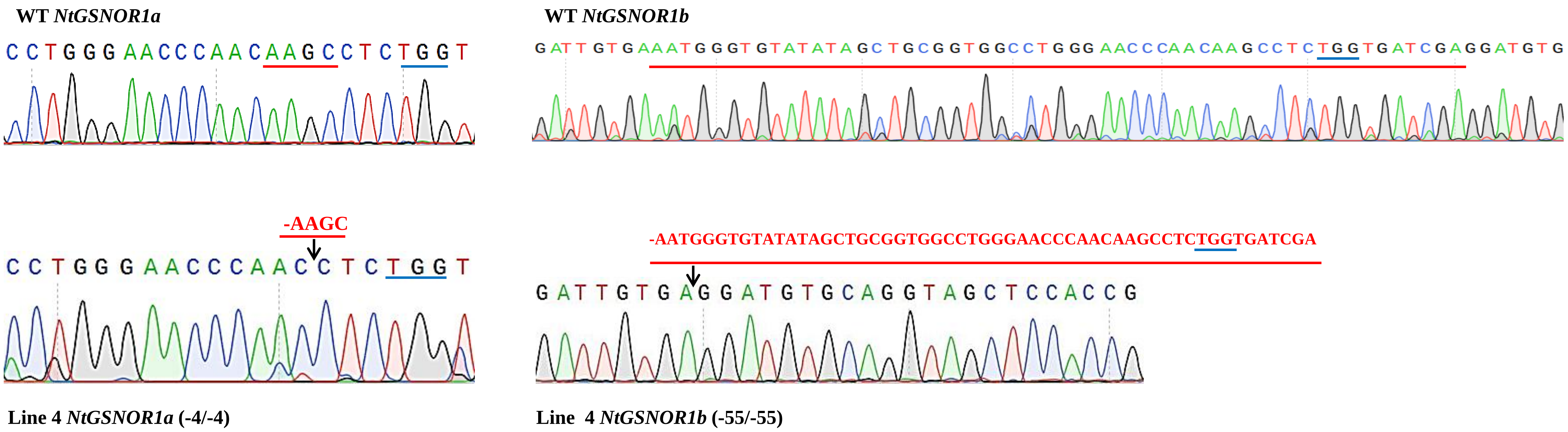

Fig. S3. Comparison of the sequencing chromatograms of *NtGSNOR1a* and *NtGSNOR1b* alleles between wildtype (top panel) and the transgenic CRISPR/CAS9 line 4 (bottom panel). The sequencing results indicated that a 4-bp sequence (AAGC) was deleted in the two alleles of *NtGSNOR1a* (bottom left) and a 55-bp sequence was deleted in the two alleles of *NtGSNOR1b* (bottom right) of the CRISPR/CAS9 line 4. The positions of the deletions are pointed by arrows and the deleted sequences are marked by red underlines in the WT chromatograms and provided on top of the chromatograms of the CRISPR/CAS9 line 3. The PAM sequence is underlined in blue.

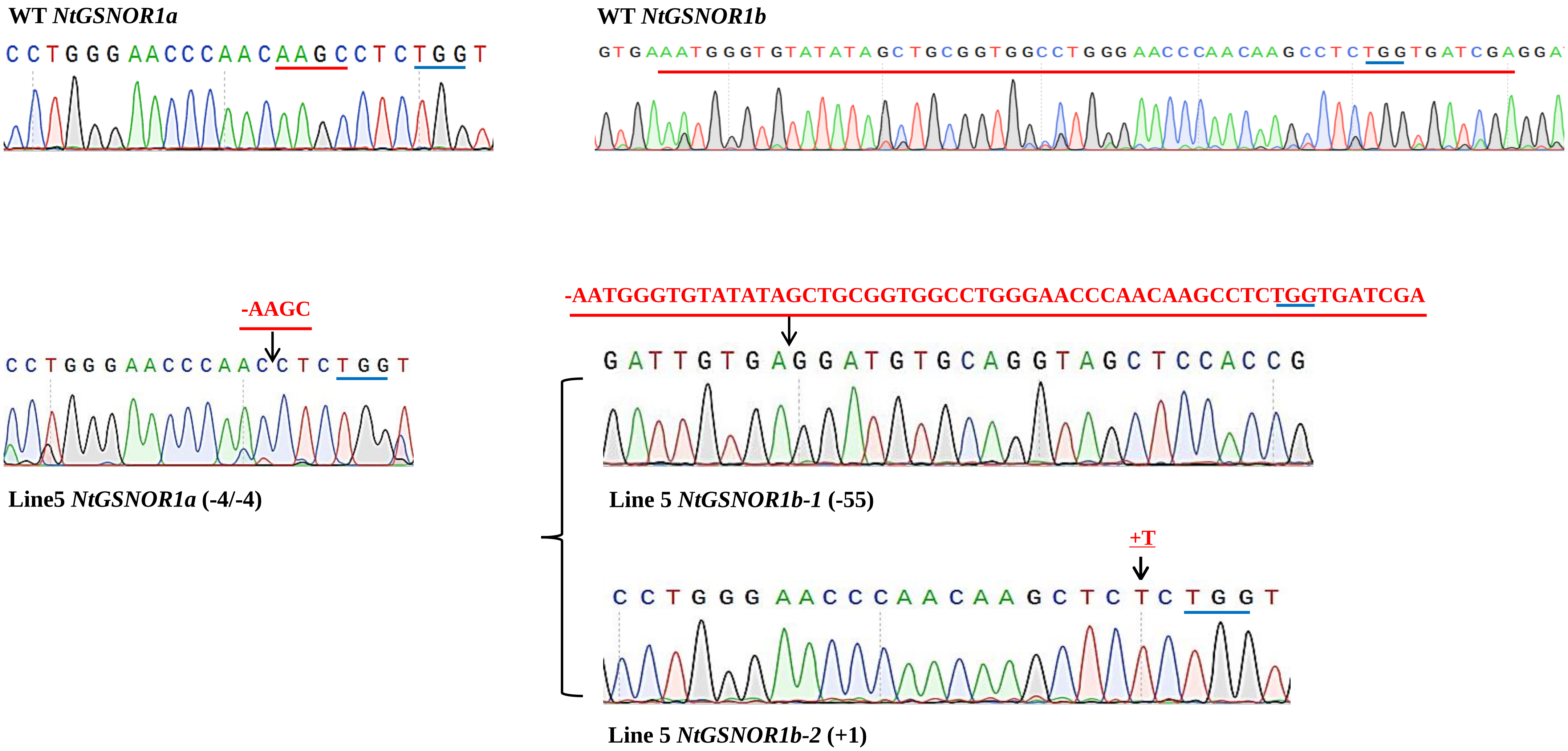

Fig. S4. Comparison of the sequencing chromatograms of *NtGSNOR1a* and *NtGSNOR1b* alleles between wildtype (top panel) and the transgenic CRISPR/CAS9 line 5 (bottom panel). The sequencing results indicated that a 4-bp sequence (AAGC) was deleted in the two alleles of *NtGSNOR1a* (bottom left). A 55-bp sequence was deleted in one of the alleles the *NtGSNOR1b* (bottom right, upper panel) and a T was inserted in the other allele of the *NtGSNOR1b* (bottom right, lower panel) of the CRISPR/CAS9 line 5. The positions of the deletions or addition are pointed by arrows and the deleted sequences are marked by red underlines in the WT chromatograms and provided on top of the chromatograms of the CRISPR/CAS9 line 5. The PAM sequence is underlined in blue.

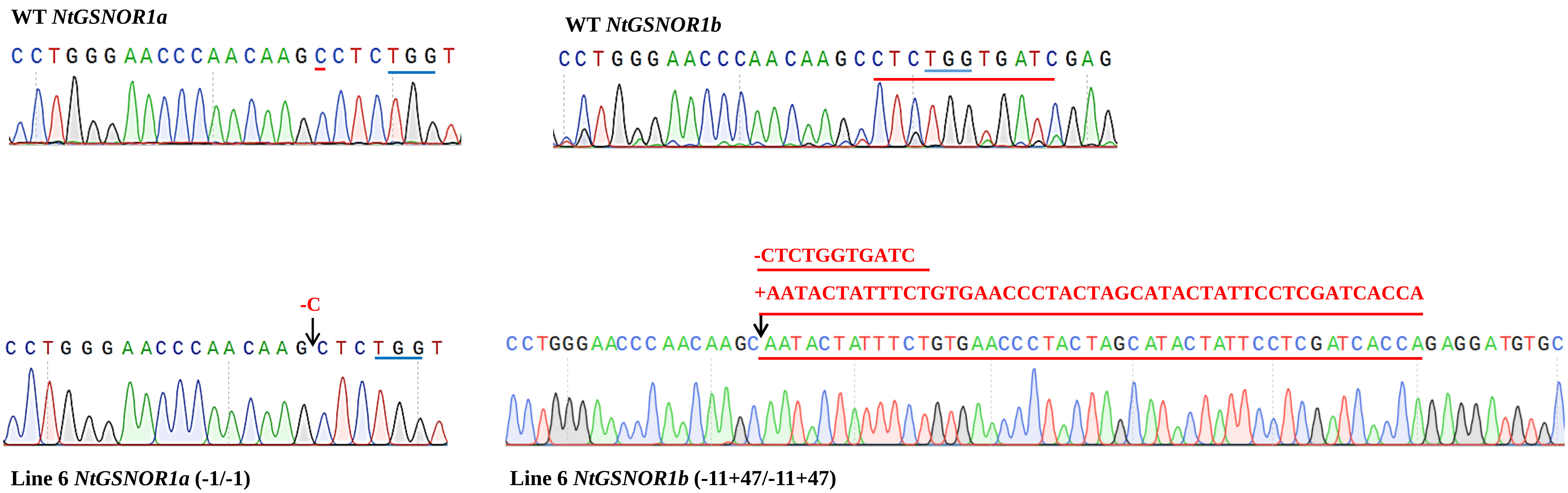

Fig. S5. Comparison of the sequencing chromatograms of *NtGSNOR1a* and *NtGSNOR1b* alleles between wildtype (top panel) and the transgenic CRISPR/CAS9 line 6 (bottom panel). The sequencing results indicated that a C was deleted in the two alleles of *NtGSNOR1a* (bottom left). A 11-bp sequence (CTCTGGTGATC) was deleted but a 47-bp sequence was inserted right after the deleted position of the *NtGSNOR1b* alleles of the CRISPR/CAS9 line 6 (bottom right). The positions of the deletions are pointed by arrows and the deleted sequences are marked by red lines in the WT chromatograms and provided on top of the chromatograms of the CRISPR/CAS9 line 6. The inserted sequence was underlined in red line in the chromatogram of the *NtGSNOR1b* alleles. The PAM sequence is underlined in blue.

CRISPR/CAS9 Knockout lines

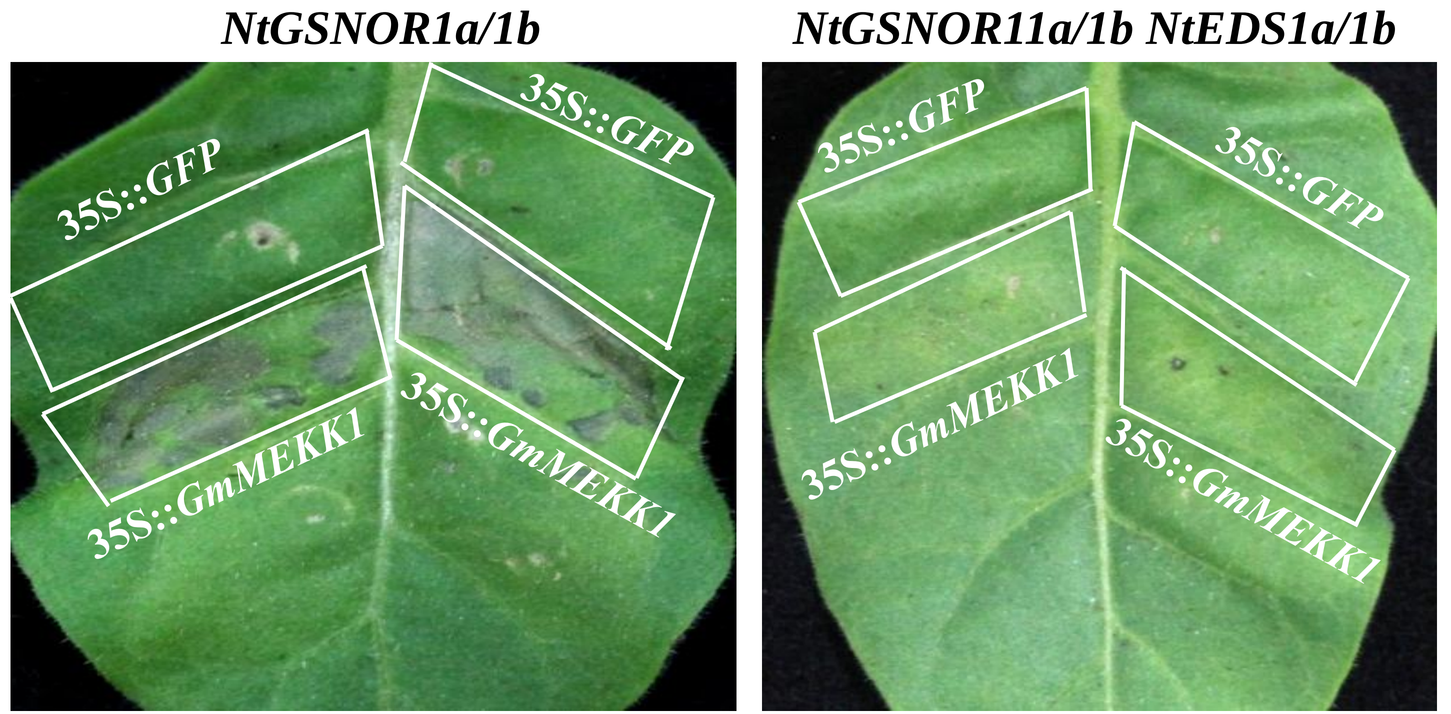

Fig. 6S. The cell death induced by transiently over-expresing *Gm*MEKK1 is abolished in *NtGSNOR1a/1b* + *NtEDS1a/1b* knockout line. The agrobacterial solution of GV3101 strain carry a 35S::*GmMEKK1* binary vector was infiltrated onto the *NtGSNOR1a/1b* knockout plants and the *NtGSNOR1a/1b* + *NtEDS1a/1b* double knockout plants, respectively. The cell death induced by over-expressing *Gm*MEKK1 (Xu et al., 2018) was observed on the leaves of the *NtGSNOR1a/1b* knockout line (left image). However, the *GmMEKK1*-induced cell death was abolished on the leaves of the *NtGSNOR1a/1b* + *NtEDS1a/1b* double knockout plants (Right image). These results indicated that *NtEDS1a/1b* was indeed knocked out in the double knockout line. The GV3101 strain carring the 35S::*GFP* construct was infiltrated as a negative control.

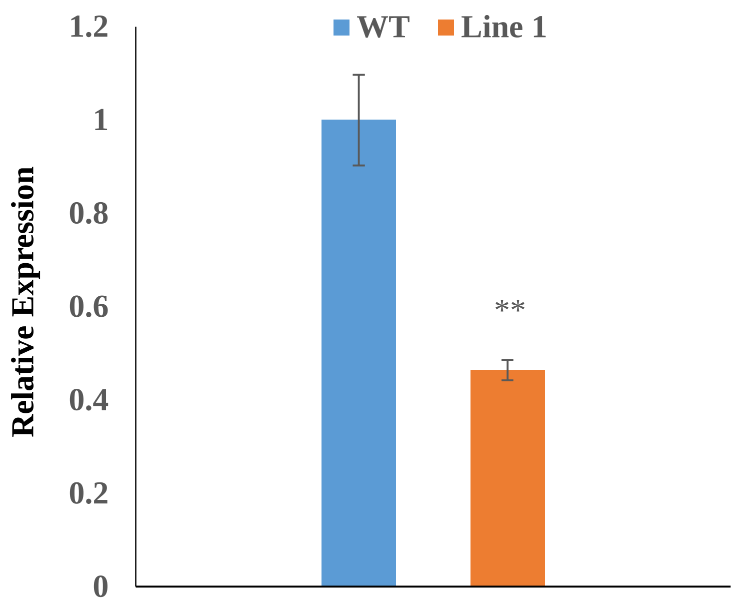

Fig. S7. The transcript level of the TMV capsid protein (CP) was reduced in the TMV-infected *NtGSNOR1a/1b* knockout plants (Line 1) relative to the TMV infected wildtype (WT) plants. The leaf discs, each containing a single HR from both the TMV-infected *NtGSNOR1a/1b* knockout plants and the TMV infected wildtype plants at 5-day post inoculation (dpi) were collected by a puncher with a diameter of 0.5 cm. 13 leaf discs containing the HRs were pooled and the total RNA was isolated from the pooled leaf discs. The qRT-PCR was performed subsequently using the SYBR green kits (Takara). The CP primers were used are: CP-F: GCTCTCGAAAGAGCTCCGAT and CP-R: TTTATCGCGCTCCTTATGGC. The actin gene was used as the endogenous reference gene. Error bars represent SD of 3 replications. Asterisks indicate significant differences from the WT control plants (**, $P<0.001$, Student's t-test).
